# The effect of the Hambisela programme on stress levels and quality of life of primary caregivers of children with cerebral palsy: A pilot study

**DOI:** 10.4102/sajp.v75i1.461

**Published:** 2019-02-20

**Authors:** Tamryn van Aswegen, Hellen Myezwa, Joanne Potterton, Aimee Stewart

**Affiliations:** 1 Department of Physiotherapy, School of Therapeutic Sciences, Faculty of Health Sciences, University of the Witwatersrand, South Africa

## Abstract

**Background:**

Caregivers of children with cerebral palsy (CP) are at risk of having high stress levels and poor quality of life (QOL) which could have a detrimental effect on themselves and their children. Taking caregivers’ well-being into consideration is therefore important when providing rehabilitation to children with CP. Interventions to mediate primary caregiver stress and QOL using an educational tool have not been tested in this population in South Africa.

**Objectives:**

The aim of this study was to determine the effect of a group-based educational intervention, Hambisela, on stress levels and QOL of primary caregivers of children with CP in Mamelodi, a township in Gauteng, South Africa.

**Method:**

Eighteen primary caregivers of children with CP participated in a quasi-experimental pretest–post-test pilot study. Hambisela, a group-based educational intervention, was carried out once a week over 8 consecutive weeks. Caregiver stress and QOL were assessed before and after the intervention using the Parenting Stress Index-Short Form (PSI-SF) and the Paediatric Quality of Life-Family Impact Module (PedsQL^TM^-FIM). Sociodemographic information was assessed using a demographic questionnaire. The Gross Motor Function Classification System (GMFCS) was used to assess the gross motor level of severity of CP in the children.

**Results:**

Data were collected for 18 participants at baseline and 16 participants at follow-up. At baseline, 14 (87.5%) participants had clinically significant stress which reduced to 11 (68.8%) at follow-up. There was no significant change in primary caregiver’s stress levels (*p* = 0.72) and QOL (*p* = 0.85) after the Hambisela programme. Higher levels of education were moderately associated with lower levels of primary caregiver stress (*r* = −0.50; *p* = 0.03).

**Conclusion:**

Most primary caregivers in this pilot study suffered from clinically significant stress levels. Hambisela, as an educational intervention, was not effective in reducing the stress or improving the QOL in these primary caregivers of children with CP. Future studies with a larger sample size are needed to investigate the high stress levels of primary caregivers of children with CP.

**Clinical implications:**

Rehabilitation services for children with disabilities should include assessments to identify caregivers with high stress levels. Holistic management programmes should also include care for the carers.

## Introduction

Parenting a child can be stressful (Abidin 1995). The characteristics of the caregiver and child, the parent–child relationship, socioeconomic factors as well as cultural contexts can affect the caregiver’s levels of stress (Raina et al. 2005). Parenting stress has been reported to impact the parent’s relationship with the child and can be detrimental to the child’s development and well-being (Golfenshtein, Srulovici & Medoff-Cooper 2015).

Caring for a child with a chronic condition, such as cerebral palsy (CP), may be more stressful than caring for a child without a disability (Cousino & Hazen 2013; Pinquart 2018). There are conflicting findings regarding the stressors that parents of children with CP experience. Evidence as to whether the level of severity of CP has an influence on parenting stress is contradictory. Some studies have found that severe CP is associated with higher levels of parenting stress (Lach et al. 2009; Plant & Sanders 2007), while others have failed to find any association between the level of severity and parenting stress (Dehghan et al. 2016; Parkes et al. 2011). The severity of disability is a broad construct based on multiple interrelated factors that are used interchangeably in the literature. Some studies refer to severity in terms of physical disability (using the Gross Motor Function Classification System [GMFCS]), and others in terms of child behaviour or cognitive problems (Pinquart 2018; Pousada et al. 2013). Findings are inconclusive as to whether it is the child’s behaviour that adds to the stress of the parent or the parent’s stress that elicits poor behaviour from the child (Pinquart 2018; Pousada et al. 2013). Cognitive dysfunction of the child has been found to negatively impact caregivers of children with chronic disabilities (Parkes et al. 2011; S˛ims˛ek et al. 2014). Additional factors associated with increased parenting stress may include poor social support and lower levels of caregiver education (Cousino & Hazen 2013; Ong et al. 1998; Parkes, Sweeting & Wight 2015).

A simple definition of quality of life (QOL) is ‘an overall assessment of well-being across various broad domains’ (Bjornson & McLaughlin 2001:183). The domains of QOL include health, emotional, cognitive, social well-being and the impact on family functioning (Isa et al. 2013; Vila et al. 2003). Caregivers of children with CP may have lower levels of QOL than the general population (Dehghan et al. 2016; Guillamón et al. 2013; Parkes et al. 2011). Caring for children with physical impairments can be demanding on their caregivers as they depend on the carer for most activities of daily living and often this role is a fulltime commitment (Pousada et al. 2013). Preliminary results suggest that parenting stress and QOL are interrelated, so it appears that decreasing stress could improve QOL (Lee et al. 2009).

A key component in combating low levels of QOL and high levels of stress is by addressing self-efficacy (Pousada et al. 2013). Self-efficacy can be explained as the ‘sense of competence and personal control over the care situation’ (Guillamón et al. 2013:1580). Self-efficacy can determine how one examines a situation and how one copes with negative thoughts and feelings that are generated by caregiving activities (Márquez-González et al. 2009). It is the ability to ask for respite when needed and to have confidence in daily caregiving tasks. Self-efficacy is used as a coping mechanism for caregiving (Guillamón et al. 2013). By addressing self-efficacy one can empower caregivers of children with CP, which may have a positive effect on QOL and stress (Guillamón et al. 2013).

In Iran and India, caregivers’ knowledge of what CP is and how to care for children with CP have been found to be inadequate (Alsadat et al. 2013; Karande, Patil & Kulkarni 2008), and caregivers expressed a need for information, counselling and social support regarding their child’s condition (Higginson & Matthewson 2014; Sen & Yurtsever 2007). A recent study in Egypt reported that caregivers’ knowledge of CP is unsatisfactory, and an educational programme significantly improved knowledge levels (Hashem & Aziz 2018). Training programmes using video, role play and written material have been found to be effective in educating caregivers (Higginson & Matthewson 2014).

Golfenshtein, Srulovici and Deatrick (2016) reviewed interventions used to reduce stress in families with chronic conditions of childhood. No interventions for CP were included in this integrative review, and insufficient research is available on the effect of educational interventions on stress levels and QOL of primary caregivers of children with CP (Karande et al. 2008; Whittingham, Wee & Boyd 2011). Thus, the aim of this study was to determine if the Hambisela educational programme reduces stress levels and improves QOL of the primary caregivers of children with CP, and to determine factors associated with stress and QOL.

## Methods

A sample of convenience of primary caregivers of children with CP was recruited from the Baby Therapy Centre. Only caregivers who agreed to participate in the study completed the outcome measures. The GMFCS was obtained from patient records. A minimum pragmatic sample size of 16 was decided upon, as at least eight participants and no more than 10 participants are required to run the Hambisela programme. Two 8-week programmes were included in this study. This sample size is not adequately powered for statistical analysis as this was a pilot study.

Participants were included if they were primary caregivers of a child with CP, literate in English and were from Mamelodi. Potential participants who had previously undertaken the Hambisela training or who belonged to another support group were ineligible for inclusion. All outcome measures were administered in English and a translator was available to assist with any language difficulties.

### Outcome measures

The Paediatric Quality of Life-Family Impact Module (PedsQL^TM^-FIM) was used to assess QOL before and after participation in the Hambisela training programme. The PedsQL^TM^-FIM is a self-administrated questionnaire consisting of 36 items over eight categories reporting on physical, emotional, social and cognitive functioning, communication and worry. It measures parent-reported family daily activities and family relationships, contributing to an overall assessment of QOL. Items are scored on a 5-point Likert scale with scores ranging from 0 (never) to 4 (almost always). Items are reverse scored and linearly transformed to a 0–100 scale. Higher scores are indicative of better QOL. There are a further two sections: the Parent Health-Related Quality of Life (HRQL) Summary score and the Family Functioning Summary score. The internal consistency reliability between items using the intraclass correlation coefficient (ICC) is excellent (*ICC* = 0.97) and the PedsQL^TM^-FIM is valid for families with children with complex chronic health conditions including CP (Varni et al. 2004). It has not been validated in South Africa before, but the PedsQL Inventory has been used for research purposes (Scott, Ferguson & Jelsma 2017; Sherr et al. 2016). Primary caregivers with low QOL scores were referred to a social worker.

The Parenting Stress Index-Short Form (PSI-SF) was used to assess stress levels before and after the intervention and is divided into three subscales: Parental Distress (PD), Parent–Child Dysfunctional Interaction (PCDI) and the Difficult Child (DC). Each subscale consists of 12 statements. The respondents are requested to respond on a Likert scale from 1 (strongly agree) to 5 (strongly disagree). The scores are then added together to form a score for each subscale, which are then added together to give a Total Stress Score. Scores are converted into percentiles, and scores between the 15th and 80th percentile are considered normal. Scores at or above the 85th percentile are considered high levels of stress and those above the 90th percentile are considered clinically significant levels of stress. Caregivers with clinically significant stress levels were referred to the local psychologist at the Itsoseng Clinic in Mamelodi East for free counselling.

The PSI-SF has good test–retest reliability with Pearson correlations of 0.84 for the Total Stress Scores and 0.68–0.85 for subscale scores (Abidin 1995). The PSI-SF has not been validated in South Africa, but it has previously been used in a South African population and was found to be reliable (Potterton 2007; Smith et al. 2017).

The PedsQL Family Information Form was used to collect information on employment and marital status, parent and child age, and relationship and information of the child’s health over the last 12 months. The severity of gross motor dysfunction of the children was established using the GMFCS, obtained from the patient’s records. The GMFCS is internationally recognised as a valid and reliable way of classifying the level of gross motor disability (Gunel et al. 2009).

Hambisela is a training and development programme aimed at providing therapeutically correct skills for caregivers of children with CP. It aims to increase the knowledge of CP as a condition and provides caregivers with therapeutically correct skills to improve daily activities such as handling, feeding, communication, dressing and play (Cerebral Palsy Eastern Cape, SA 2008). The programme consists of learning modules and practical sessions. The sessions are led by an experienced therapist, and the focus is on learning from each other’s personal experiences. The programme is known to have been used in a previous qualitative study (Djobo 2015).

The training took place over 8 consecutive weeks. The programme ran weekly for 3 h per session. After each session a manual was given to each participant summarising the topic discussed (Table 1). Participants were sent a cell phone text message the day before each session to remind them of the following day’s training session. Participants were reimbursed for their transport costs for the duration of the programme and were provided with tea and snacks during each session.

**TABLE 1:** Hambisela programme content.

Week	Theme	Contents	Materials used
1	Introduction	Discusses the definition of CP and the causes of CP. It furthermore explains associated problems and how the brain influences movement and posture. Participants are provided with opportunities to share their experiences about how they found out their children had CP.	Videos, pictures and group discussions.
2	Development	Provides an explanation of normal development. This theme furthermore explains how a parent may identify whether their child is developing typically by using a development chart. Participants are provided with information about milestones that can be expected in their own children in the future. The participants are provided with group tasks about typical development.	Pictures and charts.
3	Positioning	A practical session that teaches the participant how to position their child as well as how one may show others to position their child optimally. Participants assume the positions themselves to feel what an uncomfortable position is and how to change it. Equipment to enhance positioning is discussed.	Pictures and practical demonstrations.
4	Communication	Provides participants with an understanding of communication and the importance thereof. Participants are informed about what to do to assist their child to communicate. Various methods of communication are discussed and an emphasis is placed on finding other ways to communicate besides talking such as signing, using communication boards and reading body language.	Pictures, activities, practical demonstrations and group discussions.
5	Everyday activities	Explains how participants may use everyday activities such as bathing to assist their child to develop optimally.	Role play, activities, group discussions.
6	Feeding	Provides participants with an understanding of the possible feeding difficulties that children with CP have. Caregivers of children who are mainly dependent for feeding, will learn to find alternative ways to make feeding easier and more enjoyable. Caregivers of children, who are almost independent, will learn ways to make their child feed independently. This theme is a practical session in which participants practice spoon-feeding and cup drinking with partners in the group. The importance of positioning while feeding is highlighted.	Pictures, activities, practical demonstrations and group discussions.
7	Play	Explains the importance of play for a child’s development and will provide a caregiver with ideas on how to use play to promote development of communication, movement, social and emotional skills and learning.	Role play, activities, group discussions.
8	Graduation	Participants graduate from the programme and in this session they practise what they have learnt on their children and give feedback regarding their experiences.	Practical application of acquired skills and knowledge.

CP, cerebral palsy.

*Source*: Cerebral Palsy Association (Eastern Cape), 2008, *Hambisela: Towards excellence in cerebral palsy. A Training resource for facilitators, parents, caregivers and persons with cerebral palsy*, viewed n.d., from info@hambisela.co.za; www.hambisela.co.za.

Descriptive statistics, using means and frequencies appropriate to distribution, were used to analyse the demographic data, using Microsoft Excel. Within group changes over time, from baseline until after the 8-week Hambisela intervention programme, for the PSI-SF and PedsQL^TM^-FIM were calculated using the dependent *t*-test (*p* < 0.05). Statistica version 13 was used to analyse the data (StatSoft Inc, Tulsa, OK). The relationships between demographic factors, caregivers’ stress levels and QOL were analysed using a Spearman’s Rank Correlation Coefficient. All available data at each time point were analysed.

### Ethical considerations

This quasi-experimental pretest–post-test study took place at an outreach programme in Mamelodi, a township northeast of Pretoria, South Africa. The township is an under-resourced area challenged by inaccessible health care services, malnutrition and poverty (Mahajan 2014). Ethical clearance for this study was obtained from the Human Research Ethics Committee of the University of the Witwatersrand (M140708). Permission was granted by the Baby Therapy Centre to approach their patients. Written informed consent in English was obtained from the children’s legal guardians prior to their inclusion and participation in the study. Confidential storage of their personal data was assured. Participant confidentiality was maintained and the research adhered to the principles of the Declaration of Helsinki (2013).

## Results

Eighteen primary caregivers were included in the study. Two participants failed to complete the Hambisela programme and were considered as lost to follow-up (LTFU). The reasons for LTFU included illness of the child (*n* = 1) and work-related demands of the primary caregiver (*n* = 1). The programme was well attended with 84.6% of sessions attended. Mothers were the primary caregiver *n* = 17 (94.4%) in all but one instance, where the grandmother fulfilled this role. Most primary caregivers *n* = 14 (82.4%) were unemployed and the majority *n* = 11 (61%) were single. The demographic information of the caregivers obtained from the PedsQL^TM^ Family information form is presented in Table 2. The mean age of the children was 36.5 (± 6) months. The GMFCS level V (limited motor function in all areas with full dependence on activities of daily living) was the most common with 12 of the 18 (66.7%) children falling into this category. Ten (55.5%) children were between the ages of 3 and 5 years, five (27.8%) were below the ages of 2 and 3 years, three (16.7%) between the ages of 6 and 8 years. Twelve (66.7%) children were males.

**TABLE 2:** Demographic information of the caregivers and their children (*n* = 18).

Variables	Frequency (*n*)	Percentage (%)
**Caregiver**		
Marital status (*n* = 18)		
Single	11	61.0
Married	3	16.6
Living with someone	4	22.4
Educational level (*n* = 16)		
6th grade or less	2	12.5
7th – 9th grade or less	0	0.0
9th – 12th grade or less	4	25.0
High school graduate	4	25.0
Some college or certification course	3	18.8
College graduate	1	6.2
Graduate or professional degree	2	12.5
Employed (*n* = 17)		
Yes	3	17.6
No	14	82.4
**Children**		
Age in years (*n* = 18)		
0–2	5	27.8
3–5	10	55.6
6–8	3	16.6
Gross motor function classification system (GMFCS) (*n* = 18)		
I	3	16.7
II	1	5.6
III	0	0.0
IV	2	11.0
V	12	66.7

Mean caregiver age (years) = 32.1 (±5.6).

Note: Only data from fully completed questionnaires were analysed.

The Total Stress Score was compared for all primary caregivers before and after the intervention. Before the intervention 14 of the 16 (87.5%) participants had a Total Stress Score above the 90th percentile, indicating clinically significant levels of stress (Abidin 1995). After the intervention 11 (68.8%) participants presented with a Total Stress Score above the 90th percentile (*p* = 0.20). No statistically significant difference using the dependent *t*-test (*p* = 0.72) was found in the Total Stress Score as a result of the intervention. Clinically, there was a decrease in stress to below the 90th percentile in three (18.8%) of the participants.

No significant differences were found before and after the intervention in any of the stress categories as can be seen in Table 3. The Total Stress Scores were exceptionally high in this population (Table 3). Similarly, no significant differences using the dependent *t*-test were seen between the primary caregiver’s Total QOL Score before and after the intervention (Table 4). The Total Score is low, indicating a poor QOL in these participants. There was a significant difference (*p* = 0.047) in the ‘Daily Activities’ subcategory. Participants scored lower scores after the intervention, implying that these activities became more stressful.

**TABLE 3:** Parenting stress index-short form results.

Elements measured	Before		After		*p*
	Raw score Mean (SD)	Percentile score	Raw score Mean (SD)	Percentile score	
Total stress score	104.4 (19.5)	95th	102.8 (20.1)	95th	0.72
Parental distress	36.6 (11.1)	90th	36.1 (11.3)	90th	0.82
Parent–child dysfunctional interaction	33.2 (7.7)	99th	33.8 (6.7)	99th	0.76
Difficult child	35.1 (5.8)	90th	34.0 (7.1)	85th	0.51

SD, standard deviation.

**TABLE 4:** Paediatric quality of life-family impact module results (*n* = 16).

Elements measured	Before	After	*p*
	Mean (± SD)	Mean (± SD)	
Total score	55.5 (24.8)	56.3 (17.8)	0.850
Parent HRQL score	31.0 (13.9)	32.8 (11.5)	0.560
Physical functioning	60.3 (25.9)	54.4 (17.7)	0.150
Emotional functioning	56.6 (26.4)	61.3 (24.4)	0.470
Social functioning	52.7 (29.3)	57.1 (21.9)	0.650
Cognitive functioning	63.0 (31.8)	57.0 (30.4)	0.120
Family function score	56.7 (30.2)	51.3 (19.9)	0.350
Communication	59.4 (33.9)	63.5 (27.2)	0.540
Worry	49.7 (24.9)	50.6 (21.7)	0.810
Daily activities	56.1 (23.0)	46.1 (25.6)	0.047*
Family relationships	61.6 (34.8)	53.4 (20.3)	0.220

HRQL, health-related quality of life; SD, standard deviation.

* , statistical significance: *p* < 0.05.

There was no correlation between the child’s age, level of severity of impairment, caregiver’s age or employment status and level of stress. A moderate negative correlation (*r* = −0.50; *p* = 0.03) was found between the educational level and Total Stress Score, indicating that participants who had attained a higher educational level experienced lower stress levels (Table 5).

**TABLE 5:** Factors influencing primary caregiver stress and quality of life.

Variables	Parenting stress			Quality of life		
	*r*	*p*	Mean (SD)	*r*	*p*	Mean (SD)
Educational level	-0.50	0.03*	-	0.13	0.62	-
Caregiver age (years)	-0.12	0.82	32.1 (± 5.6)	-0.48	0.14	32.1 (± 5.6)
Child’s age (months)	0.22	0.42	36.5 (± 6.0)	0.22	0.42	36.5 (± 6.0)
Level of severity	0.34	0.21	4.0 (± 1.5)	-0.14	0.58	4.0 (± 1.5)

SD, standard deviation.

* , statistical significance: *p* < 0.05.

There was no correlation between primary caregivers’ educational level, employment status, child’s age, level of severity and participants’ QOL. A moderate, non-significant negative correlation (*r* = −0.48; *p* = 0.14) was found between the caregiver’s age and QOL, possibly indicating that the older the caregiver the poorer the QOL (Table 5).

## Discussion

In this pretest–post-test pilot study, we examined whether the Hambisela educational programme aimed at caregivers of children with CP had an impact on reported stress levels and QOL. No significant difference was found in caregiver stress levels before or after the intervention (*p* = 0.72). The mean Total Stress Score was exceptionally high, falling above the 90th percentile, indicating clinically significant stress. These results show higher stress levels than two previous studies in South Africa examining stress in caregivers in children with CP (Haniff et al. 2005; Pugin 2007). They are, however, comparable to South African caregivers of infants who are HIV infected (Potterton, Stewart & Cooper 2007) and caregivers of children with congenital heart disease (Smith et al. 2017). Sung and Park (2012) also found no reduction in stress levels after a 5-day-long family support programme for caregivers of children with disabilities. They inferred that stress as a result of caring for children with severe disabilities is not a measure that can easily be changed within a short time frame. In spite of not showing a significant improvement in stress levels before and after the Hambisela intervention, some change was shown, with three of the caregivers reporting reduced stress levels below the clinically significant 90th percentile.

All of the stress category scores apart from the DC category remained exactly the same before and after the intervention. The DC category dropped from the 90th percentile to the 85th percentile. Although this change was not statistically significant, it is possible that the participants began to realise that their children behave the way they do because of their condition and not because they are being difficult on purpose. The training may have changed their perception of their child’s behaviour and possibly influenced their QOL in the emotional category. A longer time frame between pre- and post-testing and a larger sample size might have given more significant results.

Similarly, there was no significant difference in primary caregivers’ Total QOL Score after the intervention. The mean Parents HRQL score (31) was low in comparison to caregivers of children with chronic illnesses and disabilities in Brazil (72.2), San Diego (83.8) and Malaysia (75) (Isa et al. 2013; Scarpelli et al. 2008; Varni et al. 2004). The PedsQL^TM^-FIM does not have a cut-off describing clinically significant poor QOL. However, low scores indicate poor QOL, and compared to other countries the parent HRQL score in our cohort was considerably low. The ‘Daily Activities’ subcategory produced a significant result (*p* = 0.047), indicating that participants experienced more of a problem with the daily activities after the intervention. This could be possible because the Hambisela programme aims at using daily activities to teach caregivers how to work therapeutically with their children and how to use daily activities such as feeding, dressing and bathing to teach their children skills. The specific category in the PedsQL^TM^-FIM asks questions such as the following: did the family activities take more time and effort? and did they find it difficult to find time to finish their other household tasks? Initially, it can take time to master these skills and hence caregivers may initially feel overwhelmed and slightly anxious about whether they are doing things correctly.

QOL is just as complex as stress and is a broad term encompassing health, emotional, cognitive and social well-being (Vila et al. 2003). It could be reasoned from anecdotal comments made by the participants that the Hambisela programme improved the participants’ knowledge about their children’s condition.

‘I came here not knowing what was wrong with my child, at the clinic they didn’t tell me anything. Then I attended Hambisela and now I can tell you why he is like this, why he is slow to develop.’ (Participant IA, female, 29 years old)

‘I learnt a lot of things…I learnt to position my son, to give my son attention, to communicate with him and to hold him correctly.’ (Participant IIC, female, 44 years old)

‘I learnt a lot from Hambisela…It did a lot for me…Now I can explain to anyone what is wrong with my child.’ (Participant IE, female, 28 years old)

Other anecdotal comments suggest that the participants experienced an increase in self-efficacy:

‘I am determined, I can conquer every situation that I come across with my child.’ (Participant IE, female, 28 years old)

‘I learnt a lot…I learnt to accept my child like a normal child.’ (Participant IIB, female, 32 years old)

‘I know more about lots of things, positioning, playing with my kids and I am more confident.’ (Participant IID, female, 36 years old)

‘Hambisela helped me to love my child and other CP children because now I understand all their challenges.’ (Participant IF, female, 31 years old)

In general, participants reported a sense of belonging and an improvement in their self-esteem:

‘I am very happy to have been part of this group, I made lots of friends. The training made me realise that it is not only me that has problems with my child and I am a good mom to my child.’ (Participant IIH, female, 38 years old)

‘Hambisela taught me to look at my child in a different way, a better way.’ (Participant IID, female, 36 years old)

‘No matter the challenges we come across every day, we can face them together and united as a whole.’ (Participant IC, female, 27 years old)

Djobo’s (2015) study reported similar results: the Hambisela programme empowered mothers and changed their mentality about CP. Empowering caregivers with knowledge of their child’s condition could influence their QOL but further interventions should be included to involve their emotional and health aspects. Offering respite care services and psychotherapy could provide a more comprehensive service to improve all dimensions of QOL. An assessment of the demographic results adds to the complexity of the stress and QOL constructs.

There was no correlation between the severity of gross motor disability and Total Stress Score or QOL score. However, the majority of children were classified as level V on the GMFCS, and were completely dependent on their caregivers for everyday tasks. The sample was not equally stratified through the five different GMFCS levels and so it is not possible to determine if caregivers of children with different GMFCS classifications experience different levels of stress or QOL. There was no correlation between the child’s age and primary caregiver’s stress and QOL, nor between the primary caregivers’ stress levels and the primary caregivers’ age. Similarly, other studies have not found correlations between age of parents and stress and age of the child and QOL of the parent (Davis et al. 2010; Park et al. 2012; Plant & Sanders 2007).

The moderate non-significant negative correlation (*r* = −0.48; *p* = 0.14) between the primary caregiver’s age and their QOL found in our study possibly suggests that the QOL of the primary caregiver can be affected by their increasing age but this needs to be further explored. Because of the fact that the correlation is not significant it is difficult to draw any inferences from this result. Older parents seem to have poorer QOL than younger parents in two studies (Huang et al. 2014; Lv et al. 2009). Whether this is as a result of the aging process or the impact of parenting a child with CP needs to be further investigated.

The negative correlation (*r* = −0.50; *p* = 0.03) between the primary caregiver’s stress and level of education indicates that the higher the educational level of the participants, the lower the Total Stress Scores. Potterton et al. (2007) found that higher educated caregivers’ stress decreased over time in caregivers of children who were HIV positive. Findings by Ong et al. (1998) support this finding: the less educated the parent, the more difficult it is to have access to social, educational and medical resources. This is particularly true in developing countries as awareness and knowledge of disabilities is often lacking (Masasa, Irwin-Carruthers & Faure 2014; Sharma & Sinha 2014). Hashem and Aziz (2018) also found that educated mothers had more information and a more positive attitude compared with illiterate mothers.

Stress in this sample of caregivers of children with CP was extremely high and QOL considerably low. An education programme, such as Hambisela, was not sufficient to reduce stress or improve QOL. There appears to be a paucity of published literature describing interventions that aim to reduce stress and improve QOL in primary caregivers of children with CP. The need for these interventions is expressed in all studies describing the impact on caregivers of children with CP (Cousino & Hazen 2013; Golfenshtein et al. 2015; Higginson & Matthewson 2014; Pinquart 2018). The importance of educating caregivers about CP is clear (Andrade et al. 2017; Hashem & Aziz 2018), but more comprehensive interventions are required to have a positive effect on parenting stress and QOL. Interventions have been reported as successful in other populations when the interventions were aimed at empowerment and skill development, targeting the parent–child relationship using cognitive and support methods and improving the child’s condition. Multicomponent interventions have shown better results than single-focus interventions (Golfenshtein et al. 2016).

This study has several limitations. A major limitation in our study is the short time between pre- and post-testing as no time was allowed for the programme to be consolidated. Results were for a single site and are most likely specific to the population and may not be generalisable to the larger population of primary caregivers of children with CP. The sample size also limited the statistical analysis. The study was insufficiently powered to be able to draw meaningful conclusions; however, it can be used to establish trends and as a basis for further studies. Although the assessment tools that were used are standardised, validated and reliable tools, they have not been validated in South Africa. It is therefore important to validate these tools in South Africa to compare results with other countries and to determine if they can be used within a South African context. A further limitation to this study is that the majority of this sample had children at one level of function, namely, level V on the GMFCS. The results may not be extrapolated to caregivers of children with CP having less severe GMFCS levels.

Anecdotally, the caregivers benefited from the study but quantitatively this was not shown. More specific tools may be useful in identifying benefits of the Hambisela programme such as self-efficacy and empowerment measurement tools. Qualitative methods, such as in-depth parent interviews, could further explore the possible benefits of the Hambisela programme. Identifying specific factors that contribute to the caregivers’ stress and poor QOL particularly in South Africa could also assist in developing an effective programme to help the caregivers of children with CP.

## Conclusion

The majority of the caregivers of children with CP in this pilot study showed clinically significant high stress levels and poor QOL as measured by the PSI-SF and the PedsQL^TM^-FIM. This is a concerning finding. Our results showed that the Hambisela programme in itself may be insufficient in addressing parenting stress or improving parent QoL.

## References

[CIT0001] Abidin R., 1995, *Parenting stress index professional manual*, 3rd edn., Psychological Assessment Resources, Odessa.

[CIT0002] Alsadat R.Z., Mehdi R., Zohreh S., Maryam M. & Masoud S., 2013, ‘A survery on cargivers’ knowldege about special caring for 1-to-5 year old children with cerebral palsy and their compliance with these practices’, *Journal of Research in Rehabilitation Sciences* 9(4), 618–628, viewed 29 May 2018, from http://www.sid.ir/En/Journal/ViewPaper.aspx?ID=410774.

[CIT0003] Andrade M.M.G. de., Sá F.E. de., Frota L.M.D.C.P., Cardoso K.V.V. & Carleial G.M.A., 2017, ‘Interventions of health education in mothers of children with cerebral palsy’, *Journal of Human Growth and Development* 27(2), 175 10.7322/jhgd.126857

[CIT0004] Bjornson K.F. & McLaughlin J.F., 2001, ‘The measurement of health-related quality of life (HRQL) in children with cerebral palsy’, *European Journal of Neurology: The Official Journal of the European Federation of Neurological Societies* 8 Suppl 5, 183–93, viewed n.d., from http://www.ncbi.nlm.nih.gov/pubmed/11851747. 10.1046/j.1468-1331.2001.00051.x 11851747

[CIT0005] Butcher P.R., Wind T. & Bouma A., 2008, ‘Parenting stress in mothers and fathers of a child with a hemiparesis: Sources of stress, intervening factors and long-term expressions of stress’, *Child: Care, Health and Development* 34(4), 530–541. 10.1111/j.1365-2214.2008.00842.x 19154554

[CIT0006] Cerebral Palsy Association (Eastern Cape), 2008, *Hambisela: Towards excellence in cerebral palsy. A Training resource for facilitators, parents, caregivers and persons with cerebral palsy*, viewed n.d., from info@hambisela.co.za; www.hambisela.co.za.

[CIT0007] Chiou H. & Hsieh L., 2008, ‘Parenting stress in parents of children with epilepsy and asthma’, *Journal of Child Neurology* 23(3), 301–306. 10.1177/0883073807308712 18182646

[CIT0008] Cousino M.K. & Hazen R.A., 2013, ‘Parenting stress among caregivers of children with chronic illness: A systematic review’, *Journal of Pediatric Psychology* 38(8), 809–828. 23843630 10.1093/jpepsy/jst049

[CIT0009] Davis E., Shelly A., Waters E., Boyd R., Cook K. & Davern M., 2010, ‘The impact of caring for a child with cerebral palsy: Quality of life for mothers and fathers’, *Child: Care, Health and Development* 36(1), 63–73. 10.1111/j.1365-2214.2009.00989.x 19702639

[CIT0010] Dehghan L., Dalvand H., Feizi A., Samadi S.A. & Hosseini S.A., 2016, ‘Quality of life in mothers of children with cerebral palsy: The role of children’s gross motor function’, *Journal of Child Health Care: For Professionals Working with Children in the Hospital and Community* 20(1), 17–26. 10.1177/1367493514540816 25027158

[CIT0011] Djobo W., 2015, ‘Lessons learnt from parents training for children with cerebral palsy provided by physiotherapists at the hospital of Syvanus Olympus, Togo’, *Physiotherapy* 101(1), e319 10.1016/j.physio.2015.03.520

[CIT0012] Golfenshtein N., Srulovici E. & Deatrick J.A., 2016, ‘Interventions for reducing parenting stress in families with pediatric conditions: An integrative review’, *Journal of Family Nursing* 22(4), 460–492. 10.1177/1074840716676083 27821622

[CIT0013] Golfenshtein N., Srulovici E. & Medoff-Cooper B., 2015, ‘Investigating parenting stress across pediatric health conditions – A systematic review’, *Issues in Comprehensive Pediatric Nursing* 14, 1–49. 10.3109/01460862.2015.1078423 26367769

[CIT0014] Guillamón N., Nieto R., Pousada M., Redolar D., Muñoz E., Hernández E. et al., 2013, ‘Quality of life and mental health among parents of children with cerebral palsy: The influence of self-efficacy and coping strategies’, *Journal of Clinical Nursing* 22(11–12), 1579–1590. 10.1111/jocn.12124 23461414

[CIT0015] Gunel M.K. & Mutlu A., 2009, ‘Relationship among the Manual Ability Classification System (MACS), the Gross Motor Function Classification System (GMFCS), and the functional status (WeeFIM) in children with spastic cerebral palsy’, *European Journal of Pediatrics* 168(4), 477–485. 10.1007/s00431-008-0775-1 18551314

[CIT0016] Haniff Z., Moola F., Mthembu D., Patel N. & Phetle K., 2005, ‘Parenting stress levels of parents of disabled children compared to parents of non-disabled children’, Unpublished research report, Univeristy of the Witwatersrand.

[CIT0017] Hashem S.F. & Aziz M.A.A.El., 2018, ‘The effect of an educational intervention for improving mothers’ care for their children with cerebral palsy’, *International Journal of Nursing Didactics* 8(4), 10–20. 10.15520/IJND.V8I04.2126

[CIT0018] Higginson J. & Matthewson M., 2014, ‘Working therapeutically with parents after the diagnosis of a child’s cerebral palsy: Issues and practice guidelines’, *The Australian Journal of Rehabilitation Counselling* 20(1), 50–66. 10.1017/jrc.2014.6

[CIT0019] Huang Y.P., Chang M.Y., Chi Y.L. & Lai F.C., 2014, ‘Health-related quality of life in fathers of children with or without developmental disability: The mediating effect of parental stress’, *Quality of Life Research* 23(1), 175–183. 10.1007/s11136-013-0469-7 23839542

[CIT0020] Isa S.N.I., Aziz A.A., Rahman A.A., Ibrahim M.I., Ibrahim W.P.W., Mohamad N. et al., 2013, ‘The impact of children with disabilities on parent health-related quality of life and family functioning in Kelantan and its associated factors’, *Journal of Developmental and Behavioral Pediatrics: JDBP* 34(4), 262–268. 10.1097/DBP.0b013e318287cdfe 23538932

[CIT0021] Karande S., Patil S. & Kulkarni M., 2008, ‘Impact of an educational program on parental knowledge of cerebral palsy’, *Indian Journal of Pediatrics* 75(9), 901–906. 10.1007/s12098-008-0160-0 18810366

[CIT0022] Lach L.M., Kohen D.E., Garner R.E., Brehaut J.C., Miller A.R., Klassen A.F. et al., 2009, ‘The health and psychosocial functioning of caregivers of children with neurodevelopmental disorders’, *Disability & Rehabilitation* 31(9), 741–752. 10.1080/08916930802354948 19736648

[CIT0023] Lee C., Hwang F., Chen C. & Chien L., 2009, ‘Parenting stress and quality of life of the caregiver and preschool child with very low birth weight’, *Family Community Health* 32(3), 228–237. 10.1097/FCH.0b013e3181ab3b6a 19525704

[CIT0024] Lv R., Wu L., Jin L., Lu Q., Wang M., Qu Y. et al., 2009, ‘Depression, anxiety and quality of life in parents of children with epilepsy’, *Acta Neurologica Scandinavica* 120(5), 335–341. 10.1111/j.1600-0404.2009.01184.x 19456304

[CIT0025] Mahajan S., 2014, *Economics of South African townships special focus on diepsloot*. A World Bank study, World Bank Group, Washington DC, viewed n.d., from http://documents.worldbank.org/curated/en/217211468302413395/Economics-of-South-African-townships-special-focus-on-Diepsloot.

[CIT0026] Márquez-González M., Losada A., López J. & Peñacoba C., 2009, ‘Reliability and validity of the Spanish version of the revised scale for caregiving self-efficacy’, *Clinical Gerontologist* 32(4), 347–357. https://doi:10.1080/07317110903110419

[CIT0027] Masasa T., Irwin-Carruthers S. & Faure M., 2014, ‘Knowledge of, beliefs about and attitudes to disability: Implications for health professionals’, *South African Family Practice* 47(7), 40–44. 10.1080/20786204.2005.10873260

[CIT0028] Ong L.C., Afifah I., Sofiah A. & Lye M.S., 1998, ‘Parenting stress among mothers of Malaysian children with cerebral palsy: Predictors of child- and parent-related stress’, *Annals of Tropical Paediatrics* 18(4), 301–307, viewed 16 June 2015, from http://www.scopus.com/inward/record.url?eid=2-s2.0-0031732286&partnerID=tZOtx3y1 9924586 10.1080/02724936.1998.11747964

[CIT0029] Park M.S., Chung C.Y., Lee K.M., Sung K.H., Choi I.H. & Kim T.W., 2012, ‘Parenting stress in parents of children with cerebral palsy and its association with physical function’, *Journal of Pediatric Orthopedics. Part B* 21(5), 452–456. 10.1097/BPB.0b013e32835470c0 22735919

[CIT0030] Parkes A., Sweeting H. & Wight D., 2015, ‘Parenting stress and parent support among mothers with high and low education’, *Journal of Family Psychology* 29(6), 907–918. 10.1037/fam0000129 26192130 PMC4671474

[CIT0031] Parkes J., Caravale B., Marcelli M., Franco F. & Colver A., 2011, ‘Parenting stress and children with cerebral palsy: A European cross-sectional survey’, *Developmental Medicine and Child Neurology* 53(9), 815–821. 10.1111/j.1469-8749.2011.04014.x 21707599

[CIT0032] Pinquart M., 2018, ‘Parenting stress in caregivers of children with chronic physical condition – A meta-analysis’, *Stress and Health* 34(2), 197–207. 10.1002/smi.2780 28834111

[CIT0033] Plant K.M. & Sanders M.R., 2007, ‘Predictors of care-giver stress in families of preschool-aged children with developmental disabilities’, *Journal of Intellectual Disability Research: JIDR* 51(Pt 2), 109–24. 10.1111/j.1365-2788.2006.00829.x 17217475

[CIT0034] Potterton J., 2007, *A longitudinal study of neurodevelopmental delay in HIV infected children*, University of the Witwatersrand.

[CIT0035] Potterton J., Stewart A. & Cooper P., 2007, ‘Parenting stress of caregivers of young children who are HIV Positive’, *African Journal of Psychiatry* 10, 210–214. 19588028 10.4314/ajpsy.v10i4.30257

[CIT0036] Pousada M., Guillamón N., Hernández-Encuentra E., Muñoz E., Redolar D., Boixadós M. et al., 2013, ‘Impact of caring for a child with cerebral palsy on the quality of life of parents: A systematic review of the literature’, *Journal of Developmental and Physical Disabilities* 25(5), 545–577. 10.1007/s10882-013-9332-6

[CIT0037] Pugin A., 2007, *The relationship between severity of cerebral palsy in children and the levels of stress experienced by their parents*, Unpublished research report, Univeristy of the Witwatersrand.

[CIT0038] Raina P., O’Donnell M., Rosenbaum P., Brehaut J., Walter S.D., Russell D. et al., 2005, ‘The health and well-being of caregivers of children with cerebral palsy’, *Pediatrics* 115(6), e626–e636. 10.1542/peds.2004-1689 15930188

[CIT0039] Scarpelli A.C., Paiva S.M., Pordeus I., Varni J.W., Viegas C.M. & Allison P.J., 2008, ‘The pediatric quality of life inventory (PedsQL) family impact module: Reliability and validity of the Brazilian version’, *Health and Quality of Life Outcomes* 6, 35 10.1186/1477-7525-6-35 18492262 PMC2412859

[CIT0040] Scott D., Ferguson G.D. & Jelsma J., 2017, ‘The use of the EQ-5D-Y health related quality of life outcome measure in children in the Western Cape, South Africa: Psychometric properties, feasibility and usefulness – A longitudinal, analytical study’, *Health and Quality of Life Outcomes* 15(1), 1–14. 10.1186/s12955-017-0590-3 28103872 PMC5248508

[CIT0041] Sen E. & Yurtsever S., 2007, ‘Difficulties experienced by families with disabled children’, *Journal for Specialists in Pediatric Nursing: JSPN* 12(4), 238–252. 10.1111/j.1744-6155.2007.00119.x 17956372

[CIT0042] Sharma R. & Sinha A., 2014, ‘A study on the awareness, beliefs, and service utilization among families of children with cerebral palsy in Jalandhar District of Punjab’, *Journal of Health and Research* 1(3), 170–175. 10.4103/2348-3334.138886

[CIT0043] Sherr L., Skeen S., Hensels I.S., Tomlinson M. & Macedo A., 2016, ‘The effects of caregiver and household HIV on child development: A community-based longitudinal study of young children’, *Child: Care, Health and Development* 42(6), 890–899. 10.1111/cch.12387 PMC6086490 27514630

[CIT0044] Şimşek I.E., Erel S., Şimşek T.T., Uysal S.A., Yakut H., Yakut Y. et al., 2014, ‘Factors related to the impact of chronically disabled children on their families’, *Pediatric Neurology* 50(3), 255–261. 10.1016/j.pediatrneurol.2013.11.012 24417936

[CIT0045] Smith R., Potterton J., Ntsiea V. & Brown S.C., 2017, ‘Effect of cardiac surgery in young children with congenital heart disease on parenting stress in central South Africa: Initial outcomes’, *SAHeart* 14, 162–169. 10.24170/14-3-2715

[CIT0046] Sung M. & Park J., 2012, ‘The effects of a family support program including respite care on parenting stress and family quality of life perceived by primary caregivers of children with disabilities in Korea’, *International Journal of Special Education* 27(3), 188–198.

[CIT0047] Varni J.W., Sherman S., Burwinkle T.M., Dickinson P.E. & Dixon P., 2004, ‘The PedsQL family impact module: Preliminary reliability and validity’, *Health and Quality of Life Outcomes* 2, 55 10.1186/1477-7525-2-55 15450120 PMC521692

[CIT0048] Vila G., Hayder R., Bertrand C., Falissard B., De Blic J., Mouren-Simeoni M.C. et al., 2003, ‘Psychopathology and quality of life for adolescents with asthma and their parents’, *Psychosomatics* 44(4), 319–328. 10.1176/appi.psy.44.4.319 12832598

[CIT0049] Whittingham K., Wee D. & Boyd R., 2011, ‘Systematic review of the efficacy of parenting interventions for children with cerebral palsy’, *Child: Care, Health and Development* 37(4), 475–483. 10.1111/j.1365-2214.2011.01212.x 21392055

